# Micro‐Computed Tomography Based Whole Block Imaging of Asthma‐Associated Airway Remodeling With *Mycobacterium avium*‐Induced Cavity Formation: 3‐Dimensional Nondestructive Analysis

**DOI:** 10.1111/pin.70074

**Published:** 2025-12-31

**Authors:** Tetsuya Tsukamoto, Yasushi Hoshikawa, Alexei Teplov, Eiko Sakurai, Yasushi Matsuda, Hisato Ishizawa, Emmy Yanagita, Kaori Ushida, Naoya Asai, Kazuyoshi Imaizumi, Yukako Yagi

**Affiliations:** ^1^ Department of Pathology & Lab Medicine Memorial Sloan Kettering Cancer Center New York City New York USA; ^2^ Division of Analytical Pathology, Oncology Innovation Center Fujita Health University Toyoake Japan; ^3^ Department of Thoracic Surgery Fujita Health University School of Medicine Toyoake Japan; ^4^ Department of Pathology Fujita Health University School of Medicine Toyoake Japan; ^5^ Department of Respiratory Medicine Fujita Health University School of Medicine Toyoake Japan

**Keywords:** airway remodeling, asthma, bronchial remodeling, merged whole block imaging, micro‐computed tomography, *Mycobacterium avium*, nontuberculous mycobacterial infection, whole block imaging

## Abstract

Asthma is a recognized risk factor for nontuberculous mycobacterial (NTM) pulmonary infections, yet the precise routes of infection and dissemination remain visually unclear. We analyzed a rare surgical case of asthma complicated by *Mycobacterium avium*–associated lung cavity formation in a young adult. Following antibiotic treatment, the patient underwent left upper segmentectomy, with successful subsequent treatment of residual lesions using amikacin liposome inhalation suspension. To precisely visualize the infection route, we utilized a three‐dimensional merged whole block imaging (WBI) technique. Fifteen formalin‐fixed paraffin‐embedded tissue blocks from the resected lung were individually scanned using a custom‐built micro‐computed tomography system, reconstructed, and combined to generate merged‐WBI. Three core merged‐WBIs revealed that the remodeled asthma‐associated airway ascended from the proximal hilar region, intertwined with surrounding epithelioid granulomatous tissue, and then descended into a subpleural cavity composed of epithelioid granulomas with central caseous necrosis containing acid‐fast bacilli. Besides the distinct airway path, the WBI delineated the distribution of minor epithelioid granulomas and calcifications. This report provides the first three‐dimensional visualization of a continuous airway route extending from the hilum to a subpleural cavity using merged‐WBI, suggesting bronchial spread as a probable mechanism of NTM dissemination in the setting of chronic airway disease.

**Clinical trial registration:** No clinical trials.

## Graphical abstract

Subpleural cavities are characteristic of *Mycobacterium avium* infections, but the precise route of infection remains unclear. Using merged micro‐computed tomography of multiple formalin‐fixed paraffin‐embedded (FFPE) blocks (merged whole block imaging), we visualized a continuous airway path from the hilum to a subpleural cavity. Asthma‐associated bronchial remodeling facilitated airway extension into granulomatous tissue, suggesting a structural mechanism for mycobacterial trapping and airborne spread.

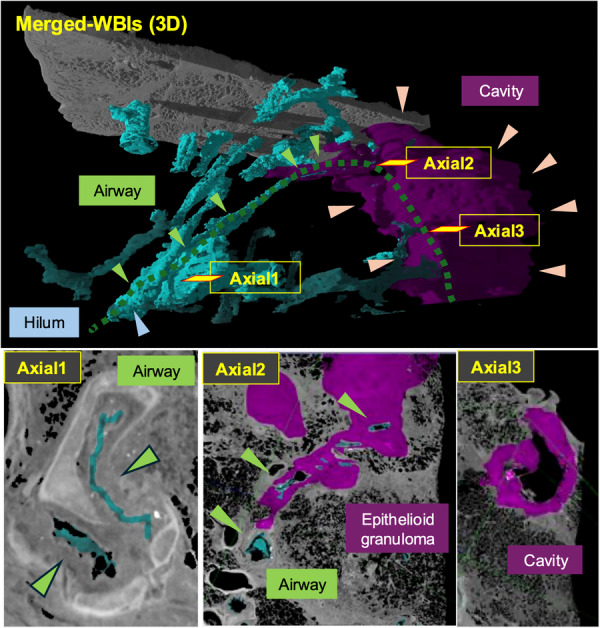

**Abbreviations**
CT: Computed tomography
WSI: Whole slide image (imaging)
WBI: Whole block image (imaging)
NTM: Nontuberculous mycobacterial
FFPE: Formalin‐fixed paraffin embedded tissue blocks
ICS: Inhaled corticosteroid
MAC: *Mycobacterium avium* complex
COPD: Chronic obstructive pulmonary disease
FEV1.0%: Forced expiratory volume % in 1 s
ALIS: Amikacin liposome inhalation suspension

## 1 Introduction

Nontuberculous mycobacterial (NTM) infections frequently occur in patients with structural lung diseases, including chronic obstructive pulmonary disease and fibrocavitary processes, as well as immune‐related conditions such as asthma [1, 2, 3]. However, it has not been fully clarified how such structural abnormalities in lung architecture and host responses may contribute to bacterial colonization and lead to disease development [1]. To explore the infection routes and patterns of bacterial colonization, we analyzed a rare surgical case of asthma complicated by *Mycobacterium avium*–associated cavity formation.

We employed the micro‐computed tomography (micro‐CT) technique to scan whole formalin‐fixed paraffin‐embedded (FFPE) tissue blocks [4] and reconstructed three‐dimensional (3D) whole block images (WBIs). Multiple WBIs were then combined to generate merged‐WBIs, providing a comprehensive overview of the lesions spanning multiple tissue blocks. This approach facilitated the visualization of airway trajectories penetrating several FFPE lung blocks, in combination with conventional two‐dimensional whole slide images (WSI).

## 2 Clinical Summary

**Figure 1:**
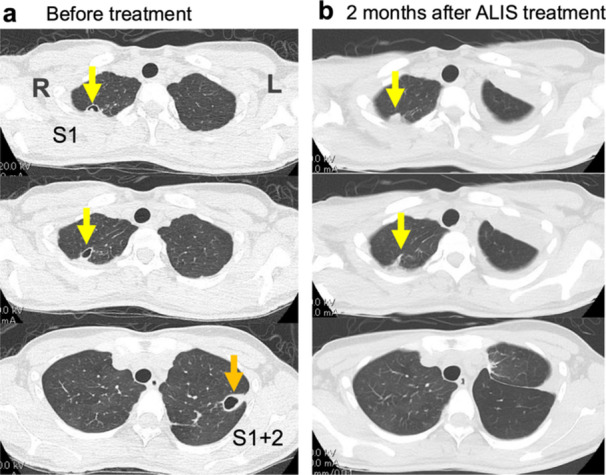
Chest CT images. (a) Pre‐treatment scan showing bilateral cavitary lesions. (b) CT scan 2 months after amikacin liposome inhalation suspension (ALIS) treatment, following segmentectomy of left upper lobe. Yellow arrow: Right upper lobe cavity showing marked alleviation with ALIS therapy. Orange arrow: Left upper lobe cavity.

The patient, an Asian woman in her 20 s, was pointed out lung abnormalities at the workplace health checkup and referred to Department of Respiratory Medicine, Fujita Health University Hospital. Chest CT exhibited thick‐walled cavities in the right S^1^ and left S^1+2^ lung segments (Figure 1a). Sputum culture confirmed the presence of *Mycobacterium avium*. The patient had a history of asthma since infancy. During the antibiotic treatment, asthma exacerbated and showed airflow limitation (Percent predicted Forced Expiratory Volume in 1 s, FEV1.0% = 57.8%), although blood eosinophils were 2.9% being within normal range (0.4–8.6%).

**Figure 2:**
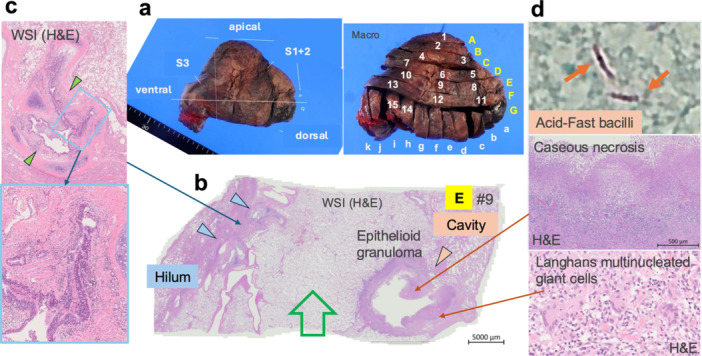
Macroscopic and representative histological findings of the resected lung specimen. (a) Macroscopic view of the excised lung with annotated block/section numbers. (b) Loupe image of section #9, showing the central region of the cavity. The hilar region is located on the left side and the peripheral cavity on the right. No airway connecting the hilum and the cavity is observed in this section (Green arrow). (c) Bronchial remodeling characterized by chronic inflammation, fibrosis, smooth muscle hyperplasia, and epithelial hyperplasia. (d) Langhans multinucleated giant cells are present in the cavity wall. Acid‐fast bacilli are visualized with the Ziehl‐Neelsen (acid‐fast) staining. Caseous necrosis lines the luminal surface of the cavity.

Treatment was started with oral antibiotics of RE‐CAM (clarithromycin 800 mg/day, rifampicin 450 mg/day, and ethambutol 750 mg/day) for 6 months. Then the patient was referred to the Department of Thoracic Surgery. Chest CT revealed the abnormal shadow in left S^1+2^, and bronchiectasis in right S^1^, S^5^, and left S^8^. To control asthma, inhalation of long‐acting muscarinic antagonist (LAMA) + long acting β2‐agonist (LABA) and oral leukotriene receptor antagonist (LTRA) were initiated. Then, intravenous amikacin (15 mg/kg body weight/day x 3 times/week) was added. Following treatment with antibiotics, a left upper segmentectomy was performed to remove the lesions in the left upper segment (S^1+2^ and S^3^) (Figure 2a). After the lung segmentectomy, inhaled corticosteroid (ICS) was started to control asthma. To treat the Mycobacterial infection especially in the right lung, amikacin liposome inhalation suspension (ALIS) was started for this poorly controlled case. ALIS therapy was discontinued following marked reduction of right S^1^ cavity after 2 years (Figure 1b).

## 3 Pathological Findings

### 3.1 Histopathological Analysis

The resected lung specimen was fixed in 10% neutral buffered formalin, sectioned, and embedded into 15 FFPE blocks (Figure 2a). A representative loupe image is shown in Figure 2b. In the proximal hilar region, the bronchus is surrounded by hyperplastic columnar epithelial cells, thickened smooth muscle bundles, and fibrotic tissue with chronic inflammatory infiltrates, findings consistent with asthma‐associated bronchial remodeling (Figure 2c). A large cavity in the peripheral subpleural region exhibited granulomatous inflammation, characterized by multinucleated Langhans giant cells and central caseous necrosis. Ziehl‐Neelsen (acid‐fast) staining revealed acid‐fast bacilli, consistent with Mycobacterium infection (Figure 2d). However, the airway connecting the hilar region to the peripheral cavity could not be identified within this single FFPE block.

### 3.2 Airway Route (Micro CT Analysis)

**Figure 3:**
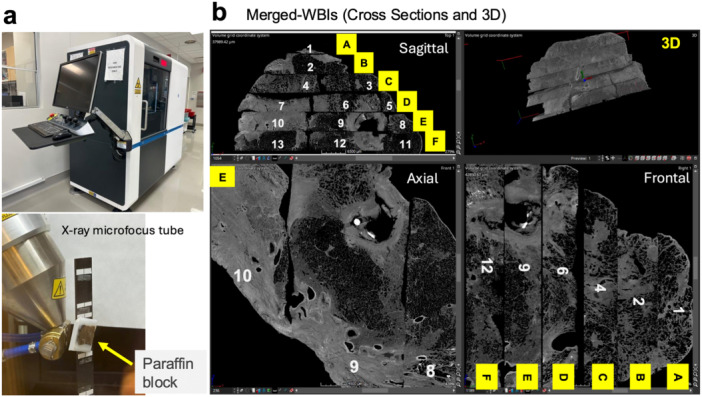
Merged whole block imaging (WBI) of 13 FFPE blocks. (a) Custom‐built micro‐CT system used for scanning (maximum resolution: 1 μm). (b) Merged‐WBIs from block #1‐#13, visualized as orthogonal cross‐sections (sagittal, axial, and frontal) and a 3D volume rendered view. The resolution of the merged‐WBI is 21 μm/voxel.

**Figure 4:**
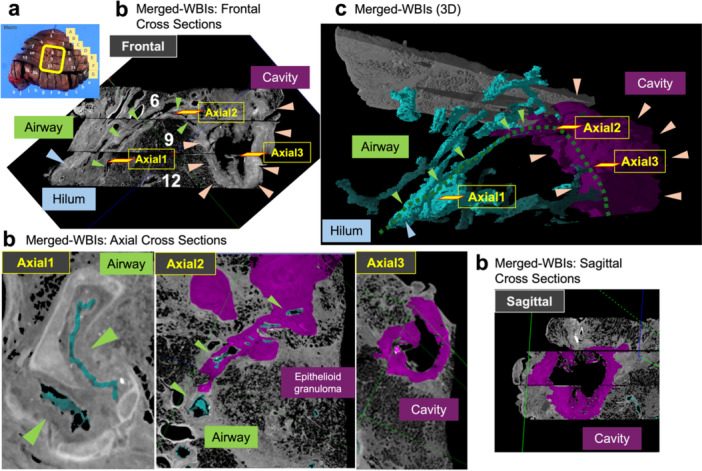
Airway route from the hilum to the subpleural cavity visualized in 2D and 3D views of the merged‐WBI from 3 FFPE blocks. (a) Location of the merged‐WBI used for imaging (yellow box). (b) Cross‐sectional images of frontal, axial, and sagittal planes. (c) 3D volume rendered reconstruction based on cross‐sectional data. The airway is annotated in cyan (green arrowhead), while the cavity (pink arrowhead) and surrounding granulomatous tissue are colored in magenta. The airway originates in the hilum (Axial1 cross‐section), ascend, and become intertwined with granulomatous tissue around the “Axial2” region. Then airway dropped into the cavity, with its central region depicted in “Axial3”. Axial cross sectional planes, Axial1, Axial2, and Axial3 in (b), correspond to respective locations in the Frontal cross section (b) and the 3D rendered view (c). Image resolution: 21 μm/voxel.

Fifteen FFPE blocks were scanned using a custom‐built micro‐CT system (3DHISTECH, Budapest, Hungary) (Figure 3a) equipped with an X‐ray microfocus tube operating at 90 kV and 6.9 W [5]. Each specimen was positioned 75.5–116.6 mm from the X‐ray source on a rotary stage, rotated 360°, and scanned at 7,835 projections per rotation with four frames per projection. The exposure time per frame was 2,000 ms, resulting in a total scanning time of 21.5 h. WBI images were reconstructed with CT Pro 3D (Nikon Metrology UK, Hertfordshire, United Kingdom) at a voxel resolution of 17–26 µm, yielding file sizes of 2.0–3.7 GB per block. Image analysis of each WBI and reconstruction of multiple merged‐WBI were performed using VG Studio MAX 2.2.6 (Volume Graphics, Heidelberg, Germany) (Figure 3b). Although the full merged‐WBI, composed of 13 WBIs (block #1‐#13), provided a comprehensive overview the lesions, the resulting file size (92.8 GB) was too large for efficient processing (Figure 3b). After reviewing the entire merged‐WBI, a reduced dataset comprising three core WBIs (blocks #6, #9, and #12; total 6.7 GB; resolution 21 μm/voxel), which encompassed the entire cavity and surrounding airways, was selected for further analysis. This subset was reconstructed separately for annotation to save computer power and memory usage (Figure 4a). Figure 4b displays frontal, axial, and sagittal cross‐sections resembling clinical CT imaging, while Figure 4c presents a 3D volume‐rendered view. The frontal section and 3D view suggest an airway originating in the hilar region, ascending, and then dropping into the subpleural cavity. Axial1 cross‐section shows the bronchus with features of airway remodeling; Axial2 indicates the airway intertwined with surrounding epithelioid granuloma; and Axial3 captures the center of the subpleural cavity.

### 3.3 Other Asthma and Mycobacterium Related Lesions

**Figure 5:**
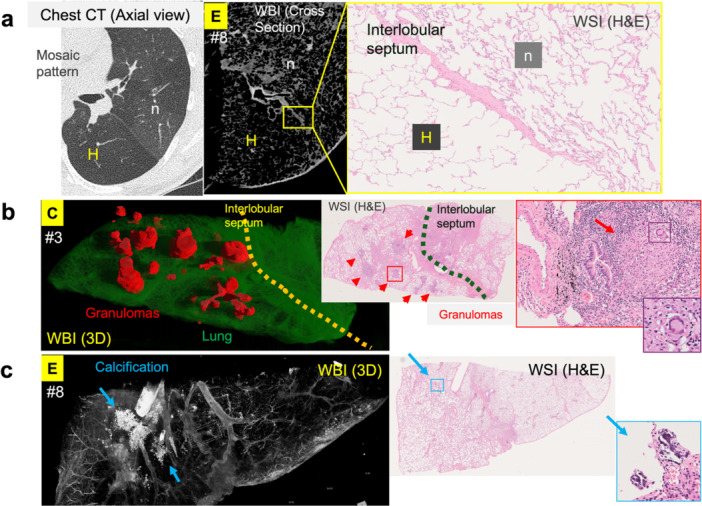
Comparison of lesions in 2D histology and 3D WBI. (a) Mosaic pattern observed on chest CT. Bottom area is hyperinflated due to air trapping by asthma‐induced bronchial remodeling. In WBI and WSI, hyperinflated and normal alveoli are clearly demarcated by the interlobular septum, confirming the chest‐CT findings. H, Hyperinflated; n: normal alveoli. (b) Small granulomas were identified in WSI of block #3. WBI revealed a greater number and volume of granulomas (annotated in red), extending throughout the left side of the interlobular septum. A thickened remodeled bronchus is visible at the center of the septum. (c) Calcification is prominently visualized in 3D WBI, whereas only faint calcified foci are detectable on the corresponding WSI.

The mosaic attenuation pattern observed in clinical CT corresponded well with alternating areas of normal and hyperinflated alveoli on both WBI and histological examination (Figure 5a). Small epithelioid granulomas were identified on the left side of the interlobular septum in WSI of block #3, whereas WBI analysis revealed a greater number and volume of granulomas exclusively on the left side of the interlobular septum, with no such lesions detected on the right side (Figure 5b). In this section, multiple granulomas situated near an artery and a bronchial tract may reflect lymphatic pathways of dissemination. In block #8, WBI demonstrated areas of calcification, although only tiny calcified foci were visible on the corresponding H&E slide (Figure 5c).

## 4 Discussion

Asthma is characterized as structural changes of airways in response to allergens or pathogens known as airway remodeling [6]. *Mycobacterium avium* complex (MAC) pulmonary disease is often associated with chronic obstructive pulmonary disease (COPD) and other structural lung conditions. It includes smoking‐related lesion followed by previous tuberculosis or granulomatous disease, radiation fibrosis, silicosis, lung cancer, bronchiectasis, chronic aspiration, and other fibrocavitary processes including asthma [1]. In the present case, histology of the proximal airway revealed established fibrosis with chronic inflammation, smooth muscle cell hyperplasia, and epithelial hyperplasia consistent with asthma associated airway remodeling. Inflammatory infiltrates composed predominantly of mononuclear cells with the absence of eosinophils might explain refractoriness to corticosteroid therapy [7]. Lack of neutrophils infiltration may also suggest a lower risk for secondary bacterial infection [8]. Fujita et al. [9] analyzed 5 surgical specimens of NTM infection, and reported histopathological features including bronchiectasis, bronchiolitis, and granulomatous lesions including cavity wall and nodules. In our case proximal bronchial alteration are likely attributable to asthma related airway remodeling as indicated in Figure 4b (Axial1 cross‐section). The subpleural cavity (Figure 4b, Axial3) appears to be formed entirely by the granulomatous inflammation by MAC infection. Notably, the intermediate region (Figure 4b, Axial2) shows a dilated airway surrounded by epithelioid granuloma, suggesting overlapping inflammation potentially driven by both asthma and MAC infection.

Fritcher et al. [2] conducted case‐control study in Canada to investigate the association between NTM infection and asthma. MAC accounted for 63.6% of the infections. Compared to controls, patients with NTM infection were significantly older (59.8 ± 8.9 vs 42.6 ± 18 years; *p* < 0.001) and exhibited severer airflow obstruction (FEV1.0%, 57% [40%–74%] vs 89.5% [80%–98%]; *p* < 0.001), who had a longer period of ICS use despite no difference in daily dosage. Hojo et al. [3] performed a nested case‐control study in Japan, in which 57.1% (8 of 14) asthmatic patients were found to have MAC infection. Those with NTM infections were older (67.1 ± 8.6 vs 58.8 ± 12.3 years, *p* < 0.01) and had severer airflow limitation (FEV1.0%, 60.6 ± 10.3 vs 72.3 ± 18.3, *p* < 0.03) than asthmatic patients without NTM infections. In contrast to the report by Fritcher et al. [2], patients in the Hojo et al. [3] study received higher doses of ICS therapy. These findings suggest that immune‐related conditions, such as asthma, particularly in older individuals with severer airflow limitation and prolonged corticosteroids exposure may increase susceptibility to NTM infection [2, 3]. In terms of treatment, ALIS combined with guideline‐based therapy has been reported to be superior to guideline‐based therapy alone in achieving negative sputum cultures in adult subjects with MAC pulmonary disease [10]. In the present case, the addition of ALIS led to marked improvement of residual right lung cavity lesions following oral and intravenous antibiotics therapy and surgical treatment.

We have previously utilized micro‐CT technique to analyze FFPE tissue blocks [4]. Xu et al. [11] demonstrated the value of this technique by identifying capsular invasion in thyroid carcinoma within the deeper region of WBIs, which was not detected in the initial H&E section. Similarly, in rectal cancer cases, Yoshida et al. [12] confirmed a connection between subserosal tumor deposit and the primary tumor mass, while Firat et al. [13] identified a tumor deposit in the circumferential adipose tissue that had been missed in the initial H&E section. Kawata et al. [14] applied micro‐CT to gastrointestinal endoscopic submucosal dissected specimens, with reconstructed 3D images enabling clear visualization of glandular structures and muscularis mucosae, thereby facilitating accurate assessment of submucosal invasion. While traditional histopathology remains advantage of precise cytological and nuclear evaluation as well as availability of special or immunohistochemical staining, routine histological sections sample only 4 μm‐thick slice from 5 mm‐thick FFPE block. Consequently, the majority of the tissue remained unevaluated. In contrast, WBIs generated by micro‐CT provide a comprehensive 3D representation of the tissue, allowing for spatial analysis of lesion distribution. In this study, WBIs were particularly valuable for quantifying the extent of granulomatous inflammation and calcification. Moreover, we potentially visualized asthma associated airway remodeling and airway route involved in NTM infection by employing merged‐WBI analysis across multiple FFPE blocks.

Although tuberculosis and NTM infection are relatively common, the precise routes of pulmonary infection remain poorly understood. The combination of caseating lesions in the hilar lymph nodes and subpleural region, referred as the Ghon focus, is a hallmark of infection and collectively termed the primary complex. However, even in standard medical textbooks, the anatomical connection between the hilar and subpleural lesions is often depicted only as a dotted line, reflecting uncertainty regarding the actual route of bacterial spread [15, 16]. Jamal and Hammer [17] classified NTM infections into two categories based on imaging findings. The first is cavitary type, typically occurs in the patients with preexisting lung diseases such as COPD or prior granulomatous disease. This type commonly spreads through bronchi and may involve the contralateral lung. It is characterized by upper‐lobe cavitary lesions accompanied by peribronchial consolidation and architectural distortion. The second type is the nodular‐bronchiectatic type, which most commonly affects the right middle lobe and lingula, often in patients with cystic fibrosis transmembrane regulator (*CFTR*) gene mutations. However, it remains unclear whether bronchiectasis serves as a predisposing factor or a consequence of the infection. In the present case, the finding could support bronchogenic spread through structurally remodeled airways, at least in part, and are consistent with the cavitary type. Our application of WBI imaging and merged‐WBI techniques provided a novel 3D visualization of this airway route, highlighting their potential utility for elucidating infection pathways, particularly in lungs with structural alterations. Potential mechanisms of Mycobacterial dissemination include direct extension, bronchial spread, or pulmonary lymphovascular routes [16]. In addition to the bronchial pathway demonstrated in this case, disseminated granulomas may also spread via lymphatic routes [18], which have not been clearly visualized using WBI. Further investigations are warranted to visualize bronchial spread of pathogens pathologically and to elucidate alternative routes of infection.

## Author Contributions

The authors confirm contribution to the paper as follows: Study conception and design: Tetsuya Tsukamoto, Yasushi Hoshikawa, Yukako Yagi; clinical data collection: Yasushi Hoshikawa, Yasushi Matsuda, Hisato Ishizawa, Kazuyoshi Imaizumi; Pathological data collection: Eiko Sakurai, Kaori Ushida, Naoya Asai; digital data preparation: Alexei Teplov, Emmy Yanagita; analysis and interpretation of results: Tetsuya Tsukamoto; manuscript preparation: Tetsuya Tsukamoto. All authors reviewed the results and approved the final version of the manuscript.

## Ethics Statement

The ethical approval and documentation for a case report was waived with approval of the Institutional Review Board at Fujita Health University, Japan. This study was conducted in accordance with the Declaration of Helsinki 1975.

## Conflicts of Interest

The authors declare no conflicts of interest.

## Data Availability

Due to patient privacy considerations, the original data supporting the results and conclusions of this study are not publicly available.

## References

[1] T. R. Aksamit , “Mycobacterium avium Complex Pulmonary Disease in Patients With Pre‐Existing Lung Disease,” Clinics in Chest Medicine 23 (2002): 643–653. 12371000 10.1016/s0272-5231(02)00022-9

[2] L. G. Fritscher , T. K. Marras , A. C. Bradi , C. C. Fritscher , M. S. Balter , and K. R. Chapman , “Nontuberculous Mycobacterial Infection as a Cause of Difficult‐to‐Control Asthma,” Chest 139 (2011): 23–27. 20829338 10.1378/chest.10-0186

[3] M. Hojo , M. Iikura , S. Hirano , H. Sugiyama , N. Kobayashi , and K. Kudo , “Increased Risk of Nontuberculous Mycobacterial Infection in Asthmatic Patients Using Long‐Term Inhaled Corticosteroid Therapy,” Respirology 17 (2012): 185–190. 21995339 10.1111/j.1440-1843.2011.02076.x

[4] M. Senter‐Zapata , K. Patel , P. A. Bautista , M. Griffin , J. Michaelson , and Y. Yagi , “The Role of Micro‐CT in 3D Histology Imaging,” Pathobiology 83 (2016): 140–147. 27100885 10.1159/000442387

[5] W. M. Sarraj , R. Tang , A. L. Najjar , et al., “Prediction of Primary Breast Cancer Size and T‐Stage Using Micro‐Computed Tomography in Lumpectomy Specimens,” Journal of Pathology Informatics 6 (2015): 60. 26730350 10.4103/2153-3539.170647 PMC4687161

[6] G. Varricchi , C. E. Brightling , C. Grainge , B. N. Lambrecht , and P. Chanez , “Airway Remodelling in Asthma and the Epithelium: on the Edge of a New era,” European Respiratory Journal 63 (2024): 2301619. 38609094 10.1183/13993003.01619-2023 PMC11024394

[7] P. Nair and F. E. Hargreave , “Measuring Bronchitis in Airway Diseases: Clinical Implementation and Application,” Chest 138 (2010): 38S–43S. 20668016 10.1378/chest.10-0094

[8] S. Pallan , J. B. Mahony , P. M. O'Byrne , and P. Nair , “Asthma Management by Monitoring Sputum Neutrophil Count,” Chest 134 (2008): 628–630. 18779198 10.1378/chest.08-0400

[9] J. Fujita , Y. Ohtsuki , I. Suemitsu , et al., “Pathological and Radiological Changes in Resected Lung Specimens in Mycobacterium avium Intracellulare Complex Disease,” European Respiratory Journal 13 (1999): 535–540. 10232422 10.1183/09031936.99.13353599

[10] A. Berlinski , “2019 Year in Review: Aerosol Therapy,” Respiratory Care 65 (2020): 705–712. 32345761 10.4187/respcare.07738

[11] B. Xu , A. Teplov , K. Ibrahim , et al., “Detection and Assessment of Capsular Invasion, Vascular Invasion and Lymph Node Metastasis Volume in Thyroid Carcinoma Using Microct Scanning of Paraffin Tissue Blocks (3D Whole Block Imaging): A Proof of Concept,” Modern Pathology 33 (2020): 2449–2457. 32616872 10.1038/s41379-020-0605-1 PMC7688566

[12] M. Yoshida , E. Cesmecioglu , C. Firat , et al., “Pathological Evaluation of Rectal Cancer Specimens Using Micro‐Computed Tomography,” Diagnostics 12 (2022): 984. 35454033 10.3390/diagnostics12040984 PMC9044748

[13] C. Firat , N. Urganci , A. Teplov , et al., “Micro‐Computed Tomography Whole‐Block Imaging Reveals Origin and Path of Rectal Cancer Tumor Deposits: A Pilot Study,” Diagnostics 14 (2024): 1704. 39202192 10.3390/diagnostics14161704 PMC11353868

[14] N. Kawata , A. Teplov , P. Ntiamoah , J. Shia , M. Hameed , and Y. Yagi , “Micro‐Computed Tomography: A Novel Diagnostic Technique for the Evaluation of Gastrointestinal Specimens,” Endoscopy International Open 09 (2021): E1886–E1889. 10.1055/a-1546-8063 PMC8670991 34917457

[15] K. M. Frank and A. J. McAdam , “Infectious Diseases.” in Robbins & Cotran Pathologic Basis of Disease, eds. V. Kumar , A. K. Abbas , and J. C. Aster (Philadelphia, PA: Elsevier, 2021). 10th edn., 339–404.

[16] F. Roberts , E. MacDuff , R. Callander , and I. Ramsden , “Respiratory System.” in Pathology Illustrated, eds. F. Roberts , E. MacDuff , R. Callander , and I. Ramsden (Philadelphia, PA: Elsevier, 2019). 8th edn..

[17] F. Jamal and M. M. Hammer , “Nontuberculous Mycobacterial Infections,” Radiologic Clinics of North America 60 (2022): 399–408. 35534127 10.1016/j.rcl.2022.01.012

[18] S. K. C. Ganchua , A. G. White , E. C. Klein , and J. L. Flynn , “Lymph Nodes—The Neglected Battlefield in Tuberculosis,” PLoS Pathogens 16 (2020): e1008632. 32790739 10.1371/journal.ppat.1008632 PMC7425845

